# An experimental in silico study on COVID‐19: Response of neutrophil‐related genes to antibiotics

**DOI:** 10.1002/hsr2.548

**Published:** 2022-03-07

**Authors:** Seyyed R. Mousavi, Hajie Lotfi, Sharareh Salmanizadeh, Sara Feizbakhshan, Farinaz Khosravian, Maryam S. Sajjadi, Sajad R. Komachali, Faeze A. Beni, Banafshe Torkan, Mohammad Kazemi, Ramin Sami, Mansoor Salehi

**Affiliations:** ^1^ Cellular, Molecular and Genetics Research Center Isfahan University of Medical Sciences Isfahan Iran; ^2^ Medical Genetics Research Center of Genome Isfahan University of Medical Sciences Isfahan Iran; ^3^ Cellular and Molecular Research Center, Research Institute for prevention of Non‐Communicable Disease Qazvin University of Medical Sciences Qazvin Iran; ^4^ Medical Genetics Laboratory Alzahra University Hospital Isfahan University of Medical Sciences Isfahan Iran; ^5^ Department of Genetics and Molecular Biology Isfahan University of Medical Sciences Isfahan Iran; ^6^ Department of Pulmonology Isfahan University of Medical Sciences Isfahan Iran

**Keywords:** ARG‐1, DEFA4, ELANE, LCN2, neutrophil‐mediated immunity, SARS‐CoV‐2

## Abstract

**Background and Aims:**

All components of the immune system are involved in alleviating severe acute respiratory syndrome coronavirus 2 (SARS‐CoV‐2) infection. Further research is required to provide detailed insights into COVID‐19‐related immune compartments and pathways. In addition, a significant percentage of hospitalized COVID‐19 patients suspect bacterial infections and antimicrobial resistance occurs following antibiotics treatment. The aim of this study was to evaluate the possible effects of antibiotics on the response of neutrophil‐related genes in SARS‐CoV‐2 patients by an experimental in silico study.

**Methods:**

The two data sets GSE1739 and GSE21802 including 10 SARS positive patients and 35 influenza A (H1N1) patients were analyzed, respectively. Differentially expressed genes (DEGs) between these two data sets were determined by GEO2R analysis and the Venn diagram online tool. After determining the hub genes involved in immune responses, the expression of these genes in 30 COVID‐19 patients and 30 healthy individuals was analyzed by real‐time polymerase chain reaction (PCR). All patients received antibiotics, including levofloxacin, colistin, meropenem, and ceftazidime.

**Results:**

GEO2R analysis detected 240 and 120 DEGs in GSE21802 and GSE1739, respectively. Twenty DEGs were considered as enriched hub genes involved in immune processes such as neutrophil degranulation, neutrophil activation, and antimicrobial humoral response. The central nodes were attributed to the genes of neutrophil elastase (ELANE), arginase 1 (ARG‐1), lipocalin 2 (LCN2), and defensin 4 (DEFA4). Compared to the healthy subjects, the expression of LCN2 and DEFA4 were significantly reduced in COVID‐19 patients. However, no significant differences were observed in the ELANE and AGR‐1 levels between COVID‐19 subjects and the control group.

**Conclusions:**

Activation and degranulation of neutrophils were observed mainly in SARS, and H1N1 infection processes and antibiotics administration could affect neutrophil activity during viral infection. It can be suggested that antibiotics can decrease inflammation by restoring the expression of neutrophil‐related genes in COVID‐19 patients.

## 1 INTRODUCTION

Coronavirus 2019 (COVID‐19) outbreaks began in the 21st century in Wuhan, Hubei Province, China, with a high transmission rate and is still a major threat to the human population due to its high mortality rate.^1^ Severe acute respiratory syndrome coronavirus 2 (SARS‐CoV‐2) is an enveloped virus with extensive single‐stranded RNA consisting of 29,903 nucleotides and belongs to Nidovirales order, Coronaviridae large family, and Coronavirinae subfamily.^2^, ^3^ The clinical manifestations of COVID‐19 vary from asymptomatic to mild symptoms of the upper respiratory tract and gastrointestinal tract to severe pneumonia with acute respiratory distress syndrome (ARDS) and death.^4^, ^5^ Innate and adaptive immune responses are essential for eliminating CoVs infected cells. The primary antiviral defense mechanism is associated with the production of interferon (IFN) Types I and III and various chemokines. These chemokines induce other innate response cells including, leukocytes, monocytes, natural killer (NK) cells, dendritic cells (DCs), and eventually recruit lymphocytes to eventually viral antigens to DCs.^6^ Based on the available findings, the inflammatory response and subsequent immunity in the early stages of COVID‐19 infection are comparable to other coronaviruses.^7^ These findings were further confirmed by increased serum levels of these molecules in COVID‐19 patients. Thus, SARS‐CoV‐2 can escape the antiviral defense system, activate the innate response, and utilize compatible immune cells. In terms of immunopathogenesis, the role of neutrophils in the development of COVID‐19 is highlighted.^8^ Neutrophils are the most abundant granulocytes involved in the innate immune system and effectively respond to various bacterial and fungal infections. The role of neutrophils in the viral defense process refers to interactions with other immune mechanisms such as cytokine release, virus internalization, and killing mechanisms.^9^ However, neutrophilia has been reported as an indicator of severe respiratory symptoms and a poor outcome in COVID‐19 patients. Moreover, significant neutrophil infiltration has been identified in COVID‐19 patients.^10^ Along with the mechanism of infiltration, the pathological effects of neutrophil extracellular traps (NETs) on various inflammatory conditions such as respiratory failure are reported. Indeed, in response to infection, neutrophils release NETs that are composed of extracellular DNA fibers, histones, antimicrobial proteins, proteases such as neutrophil elastase, and oxidant enzymes. Therefore, disruption of the regulation of these mechanisms can cause inflammation.^11^ Neutrophil elastase (ELANE), Arginase 1 (ARG‐1), Lipocalin 2 (LCN2), and Defensin 4 (DEFA4) are four vital genes involved in neutrophil‐mediated immunity. Bioinformatic studies have identified these genes as vital components that are significantly upregulated during SARS infection.^12^ Besides this, overexpression of ELANE has been determined in the nasopharyngeal swabs of COVID‐19 patients. Likewise, upregulated expression of ARG‐1 has been detected in the blood samples and nasopharyngeal aspirates of these patients as well.^13^, ^14^

In addition, dysregulated immune response to bacterial infections is an essential issue in COVID‐19 patients. Clinical evidence confirms bacterial coinfections in the hospitalized COVID‐19 patients who have previously received antibiotics.^15^

Excessive intake of antibiotics in patients with SARS‐CoV‐2 infection, which could lead to antimicrobial resistance has to be considered as well. Ultimately, the elucidation of how ELANE, ARG‐1, LCN2, and DEAF4 act as innate immunity mediators along with antibiotic effects in COVID‐19 infection can provide effective therapeutic strategies.

## 2 MATERIAL AND METHODS

### 2.1 In silico analysis

#### 2.1.1 Selection of data sets and analysis of differentially expressed genes (DEGs)

Gene Expression Omnibus (GEO) database is a free online database that provides a plethora of microarray data and gene profiles. Two microarray gene expression profiles, GSE1739 and GSE21802, were downloaded from the NCBI‐GEO database. GSE1739 represented the gene expression profile of peripheral blood mononuclear cells (PBMCs) of 10 SARS‐positive patients and four healthy individuals, which was quantified based on the GPL201 (Affymetrix Human HG‐Focus Target Array) platform.

GSE21802 contains a gene expression profile of PBMC of 35 influenza A (H1N1) positive patients and four healthy subjects.^16^ H1N1 microarray data set is assessed based on the GPL6102 Illumina human‐6 v2.0 expression bead chip. Subsequently, GEO2R online tool was applied to identify DEGs in SARS‐positive and H1N1‐positive patients compared to healthy individuals by considering |logFC| > 2 and adjusted *p* < 0.05 as cut‐off criteria. The raw data of GEO2R analysis was obtained in the TXT format and assessed by Venn diagram online tool (http://bioinformatics.psb.ugent.be) to identify common DEGs between the two mentioned data sets.

#### 2.1.2 Gene ontology and protein‐protein interaction (PPI network analysis)

To enrich the gene ontology, the hub genes were subjected to gProfiler database (https://biit.cs.ut.ee/gprofiler/)^17^ and the Cytoscape ClueGO tool.^18^ Accordingly, the analysis mode was set as functional and the virtual style was considered significant. In addition, hub genes were assessed by Cytoscape String app^19^ to predict the probable PPI of the hubs (confidence score ≥ 0.5 and interactors = 0, as the cut‐off criteria). Finally, the Cytoscape Network Analyzer tool was used to examine the interaction number of each hub and other network information.

### 2.2 Experimental analysis

#### 2.2.1 Sampling

This study was approved by the Ethics Committee of the Medical Genetics Research Center of Genome, Isfahan, Iran. Thirty COVID‐19 patients including 16 men and 14 women, with a mean age of 60.9 ± 9.8 years were enrolled from Alzahra Hospital (Isfahan, Iran). Also, 30 random samples including 13 men and 17 women with a mean age of 58.9 ± 9.4 years participated as healthy subjects for the control group. COVID‐19 patients were admitted to the intensive care unit (ICU) with severe symptoms diagnosed by an infectious disease specialist (inclusion criteria). Patients neither had a history of underlying disease nor autoimmune disorders (exclusion criteria). All patients were treated with antibiotics including Levofloxacin, Colistin, Meropenem, and Ceftazidime. Whole blood samples (5 ml) were collected in EDTA‐containing ice‐cold tubes.

#### 2.2.2 Isolation of total RNA from human whole blood

Total RNA was extracted using RNA Extraction‐Kit (Favor‐Prep, Blood/Cultured Cell Total RNA) according to the manufacturer's instructions as follows.

The red blood cells were lysed by adding whole human blood (200–300 μl) preserved with an anticoagulant to a tube. The RL buffer (5:1 ratio) was then added to each sample and was mixed with inversion followed by 10 min of incubation on ice. Samples were centrifuged at 4500 rpm for 1 min to collect cell pellets. The cell pellets were resuspended using an RL buffer followed by a vortex and further centrifuged. The supernatant was completely discarded and then FARB Buffer and β‐Mercaptoethanol were added to each sample. The samples were vigorously vortexed. The filter column was placed on a collection tube and the samples were transferred to the filter column centrifuged at 18,000*g* for 2 min. The clarified supernatants were transferred to a new tube and RNase‐free ethanol (70%) was added, mixed well, and transferred to the FARB Mini column. The samples were centrifuged at full speed and flow was discarded. The wash buffer was then added to the FARB mini‐column and centrifuged at full speed. RNase‐free DNase 1 solution (0.5 U/μl) was added to the center of the FARB mini‐column membrane. After 15 min, the washing step was carried out twice using a wash buffer, and samples were centrifuged (full speed, 1 min). The FARB mini‐column was dried to prevent liquid residue. The column was placed in a wash tube and RNase‐free ddH_2_O (40–100 µl) was added to the center of the column membrane and centrifuged at full speed for 1 min to wash the RNA. The quality of the extracted RNA was measured at a wavelength ratio of 260.280 nm using a NanoDrop spectrometer (Thermo Scientific).

#### 2.2.3 Complementary DNA (cDNA) synthesis

cDNA was synthesized using Bio‐fact standard kit (Biofact) and the process was conducted on Applied Biosystems® Veriti® 96‐Well Thermal Cycler. The following mixture was prepared in a polymerase chain reaction (PCR) tube with a total volume of 20 µl consisting of total RNA (~10 ng), random hexamer (50 µM), 2× RT Pre‐Mix, RNAase‐free water. The mixture was incubated at room temperature for 5 min followed by 30 min of incubation at 50°C to complete cDNA synthesis.

#### 2.2.4 Quantitative real‐time PCR (qPCR)

Expression of LCN2, DEFA4, ELANE, and ARG‐1 was assessed by real‐time PCR using specific primers (Table 1, Figure S1) and SYBR Green dye (Biofact) conducted on Rotor‐Gene 6000 instrument (Corbett Life Science). The qPCR results were analyzed based on the $2^{-∆∆C_{t}}$ method. Expression of glyceraldehyde‐3‐phosphate dehydrogenase was also evaluated as a reference gene.

**Table 1:** The sequence of the specific primer used in qPCR

Genes	Forward primer	Reverse primer
ARG1	5′‐TTCTCAAAGGGACAGCCACGAGGA‐3′	5′‐TTCTTGACTTCTGCCACCTTGCCA‐3′
ELANE	5′‐ACTGCGTGGCGAATGTAA‐3′	5′‐CCGTTGAGCTGGAGAATC‐3′
DEFA4	5′‐CCTTTGCATGGGATAAAAGCTCT‐3′	5′‐ACACCACCAATGAGGCAGTTC‐3′
LNC2	5′‐ACGCTGGGCAACATTAAGAGTTAC‐3′	5′‐CGATTGGGACAGGGAAGACGAT‐3′
GAPDH	5′‐AAGGTGAAGGTCGGAGTCAAC‐3′	5′‐GGGGTCATTGATGGCAACAA ‐3′

Abbreviations: GAPDH, glyceraldehyde‐3‐phosphate dehydrogenase; qPCR, quantitative real‐time polymerase chain reaction.

### 2.3 Statistical analysis

The results were statistically analyzed and normalized by Graph Pad Prism software version 8.0.2 (Graph Pad) and Shapiro–Wilk test, respectively. The unpaired *t‐test* was used to analyze and normalize the expression level between two groups according to the normally distributed genes data. All results are presented as mean ± standard deviation (SD), and *p* ≤ 0.05 was considered statistically significant.

## 3 RESULTS

### 3.1 DEGs identification

**Figure 1:**
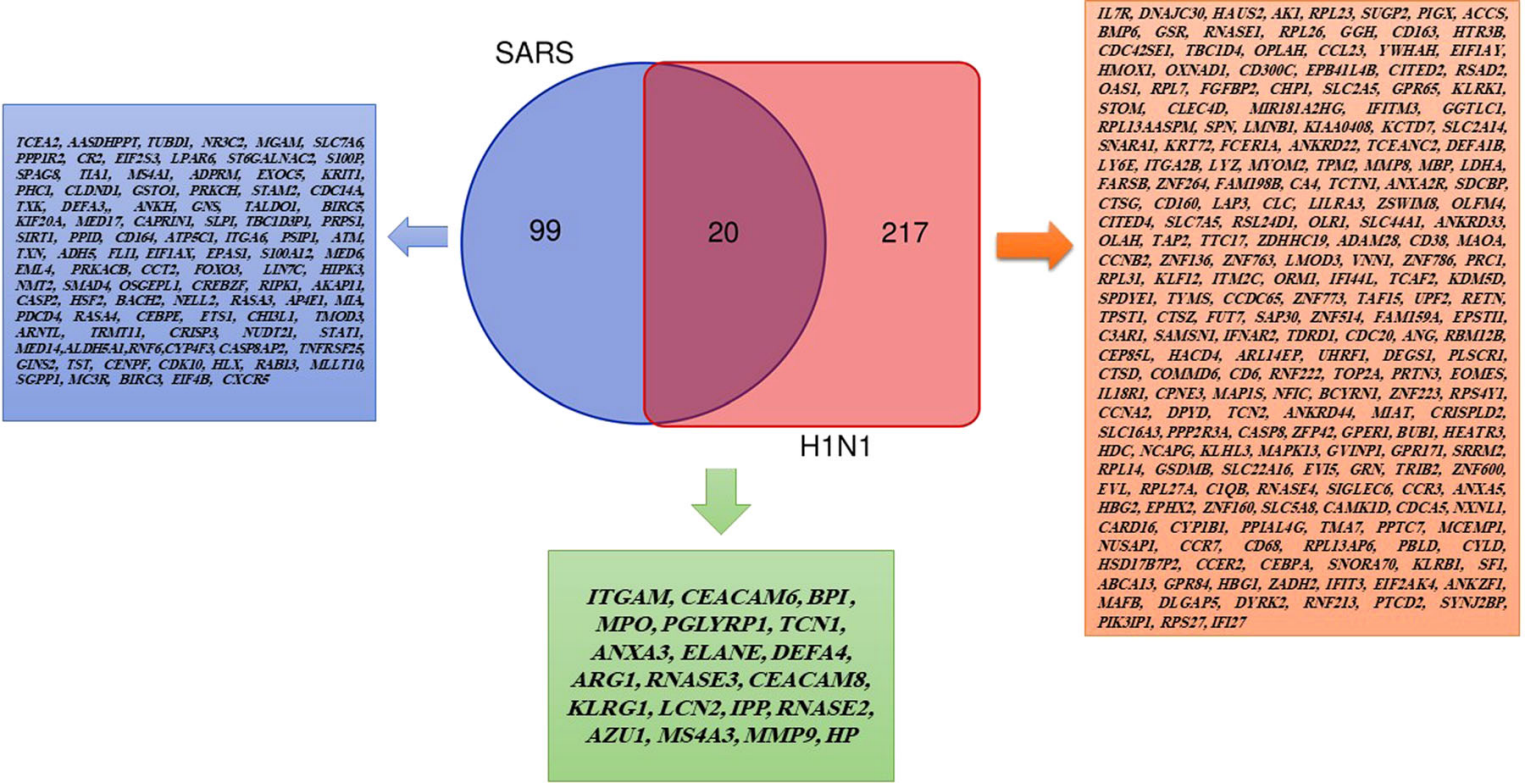
Authentication common differentially expressed genes (DEGs) between severe acute respiratory syndrome (SARS)‐positive and influenza A H1N1‐positive patients with │logFC│ > 1 and *p* < 0.05 using Venn diagram software. The green box corresponds to 20 DEGs commons between the two mentioned data sets

**Figure 2:**
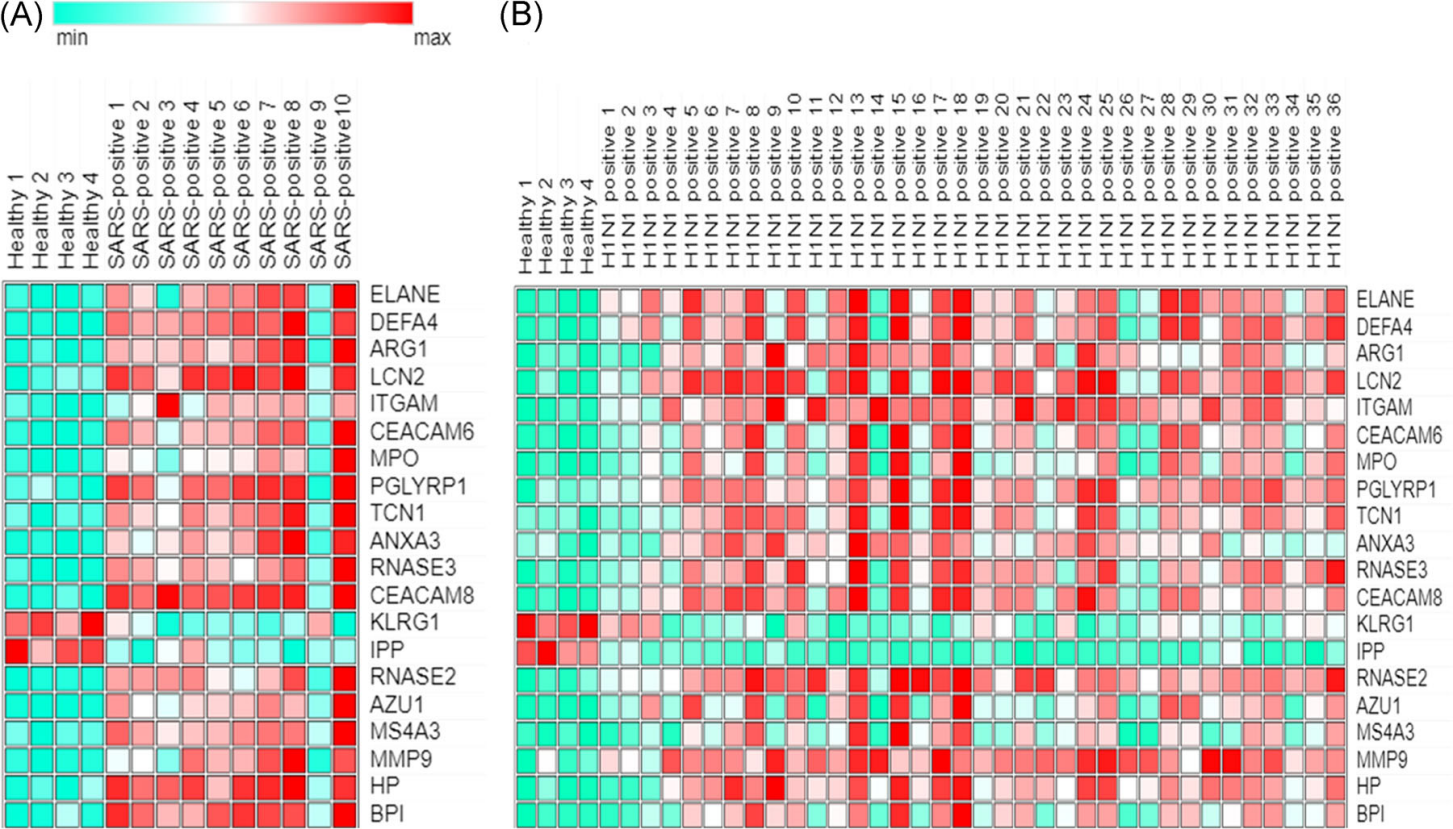
Heat map of expression of 20 hubs in (A) severe acute respiratory syndrome (SARS)‐positive and (B) influenza A H1N1‐positive samples

GEO2R analysis identified 240 and 120 DEGs in GSE21802 and GSE1739, respectively (|logFC| ≥ 1 and adjusted *p* ≤ 0.05). Next, DEGs were inputted in the Venn diagram online tool to find common DEGs between two data sets. As shown in Figure 1, including ITGAM, CEACAM6, BPI, MPO, PGLYRP1, TCN1, ANXA3, ELANE, EFA4, ARG1, RNASE3, CEACAM8, KLRG1, LCN2, IPP, RNASE2, AZU1, MS4A3, MMP9, and HPwere detected as common hubs genes between two data sets. Heat maps of the hubs in the two data sets are depicted in Figure 2.

### 3.2 Gene Ontology and PPI network analysis

**Figure 3:**
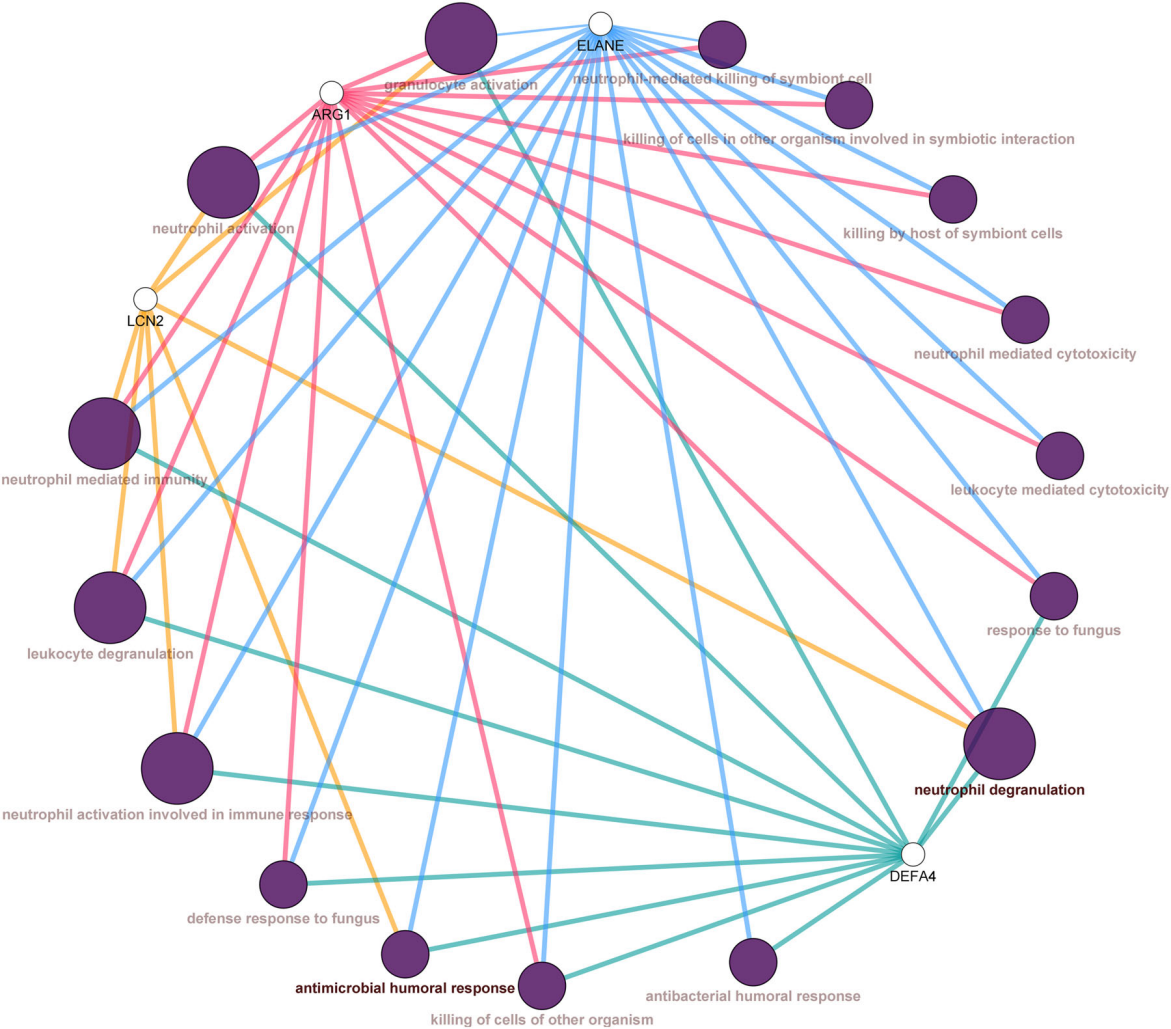
ClueGO analysis of LCN2, DEFA4, ELANE, and ARG‐1. Node sizes vary according to *p* value (≤0.05). Larger nodes represent a more significant *p* value

The gProfiler analyzing reports are listed in Table 2. According to the analysis of the ClueGO tool, the hubs were significantly enriched in crucial immune processes including neutrophil degranulation, neutrophil activation, neutrophil‐mediated immunity, leukocyte degranulation, and antimicrobial humoral response (Figure 3).

**Table 2:** Gene Ontology analysis of selected hubs in SARS and influenza H1N1 affected patients

Source	Term name	Term id	Adj *p* value	Count
GO:MF	Serine‐type endopeptidase activity	GO:0004252	0.006	4
GO:MF	Serine‐type peptidase activity	GO:0008236	0.009	4
GO:MF	Serine hydrolase activity	GO:0017171	0.010	4
GO:MF	Glycosaminoglycan binding	GO:0005539	0.023	4
GO:BP	Neutrophil degranulation	GO:0043312	3.95E‐23	17
GO:BP	Neutrophil activation involved in immune response	GO:0002283	4.40E‐23	17
GO:BP	Neutrophil‐mediated immunity	GO:0002446	6.47E‐23	17
GO:BP	Neutrophil activation	GO:0042119	6.93E‐23	17
GO:BP	Granulocyte activation	GO:0036230	8.81E‐23	17
GO:BP	Leukocyte degranulation	GO:0043299	2.53E‐22	17
GO:BP	Myeloid cell activation involved in immune response	GO:0002275	3.47E‐22	17
GO:BP	Myeloid leukocyte‐mediated immunity	GO:0002444	4.32E‐22	17
GO:BP	Myeloid leukocyte activation	GO:0002274	1.15E‐20	17
GO:BP	Leukocyte activation involved in immune response	GO:0002366	3.58E‐20	17
GO:BP	Cell activation involved in immune response	GO:0002263	3.94E‐20	17
GO:BP	Regulated exocytosis	GO:0045055	2.19E‐19	17
GO:BP	Leukocyte‐mediated immunity	GO:0002443	1.41E‐18	17
GO:BP	Exocytosis	GO:0006887	2.49E‐18	17
GO:BP	Immune effector process	GO:0002252	1.02E‐15	17
GO:BP	Leukocyte activation	GO:0045321	1.61E‐15	17
GO:BP	Secretion by cell	GO:0032940	3.00E‐15	17
GO:BP	Export from cell	GO:0140352	5.72E‐15	17
GO:BP	Cell activation	GO:0001775	1.14E‐14	17
GO:BP	Secretion	GO:0046903	1.56E‐14	17
GO:BP	Immune response	GO:0006955	1.40E‐13	18
GO:CC	Secretory granule	GO:0030141	3.14E‐19	17
GO:CC	Secretory vesicle	GO:0099503	7.27E‐18	17
GO:CC	Specific granule	GO:0042581	1.19E‐16	11
GO:CC	Secretory granule lumen	GO:0034774	3.49E‐15	12
GO:CC	Cytoplasmic vesicle lumen	GO:0060205	4.06E‐15	12
GO:CC	Vesicle lumen	GO:0031983	4.37E‐15	12
GO:CC	Primary lysosome	GO:0005766	1.43E‐14	10
GO:CC	Azurophil granule	GO:0042582	1.43E‐14	10
GO:CC	Specific granule lumen	GO:0035580	9.27E‐14	8
GO:CC	Cytoplasmic vesicle	GO:0031410	1.59E‐11	17
GO:CC	Intracellular vesicle	GO:0097708	1.63E‐11	17
GO:CC	Azurophil granule lumen	GO:0035578	3.95E‐10	7
GO:CC	Vacuolar lumen	GO:0005775	3.95E‐08	7
GO:CC	Lysosome	GO:0005764	6.05E‐08	10
GO:CC	Lytic vacuole	GO:0000323	6.05E‐08	10
GO:CC	Vesicle	GO:0031982	8.22E‐08	17
GO:CC	Vacuole	GO:0005773	2.13E‐07	10
REAC	Neutrophil degranulation	REAC: R‐HSA‐6798695	1.07E‐18	16
REAC	Innate immune system	REAC: R‐HSA‐168249	5.55E‐13	16
REAC	Immune system	REAC: R‐HSA‐168256	3.99E‐10	17

Abbreviation: SARS, severe acute respiratory syndrome coronavirus 2.

**Figure 4:**
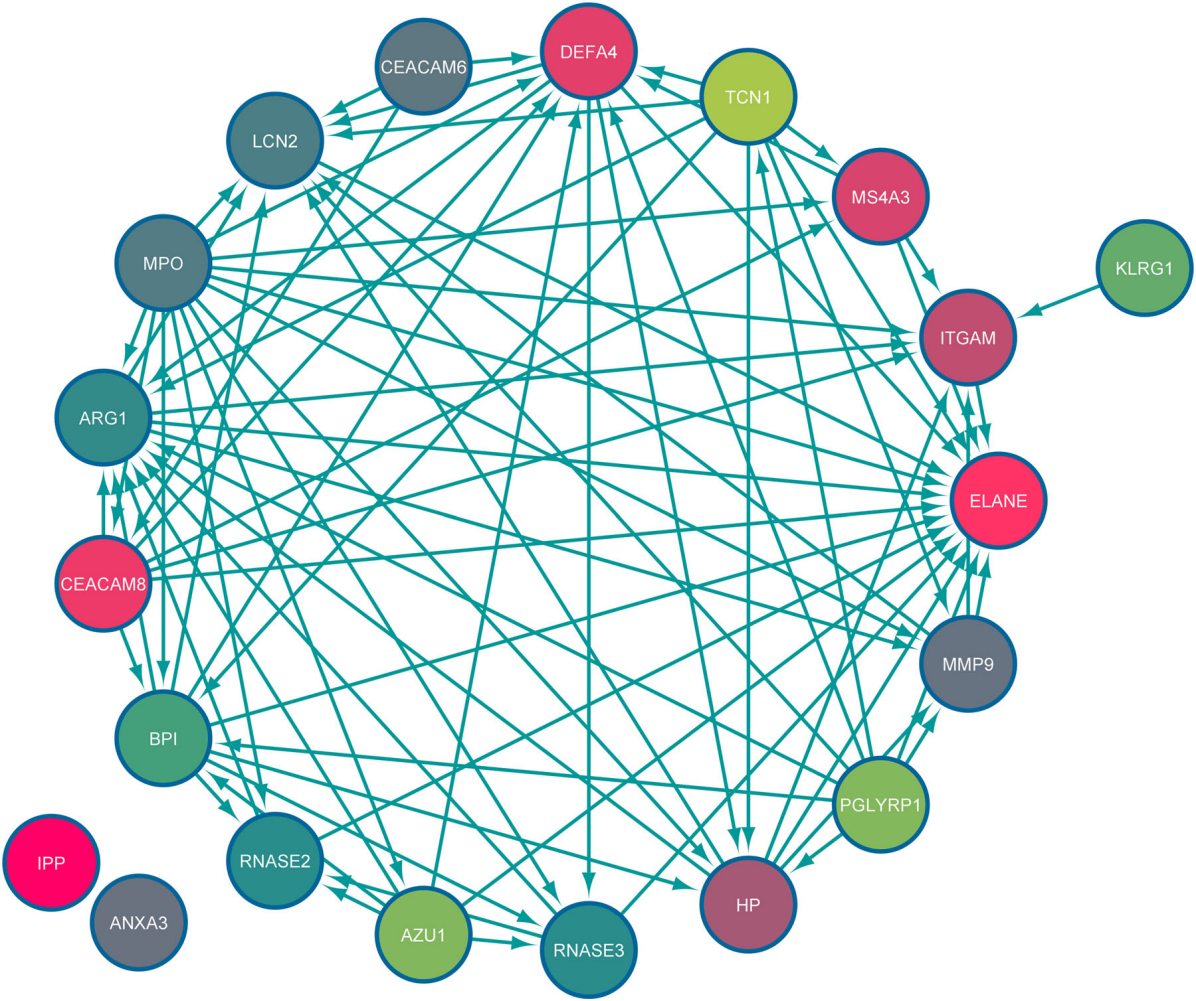
Protein–protein interaction (PPI) network analysis of hubs. Edge tags are based on scores of PPI

PPI network analysis identified the interaction/correlation between the hubs (Figure 4). In addition, network analysis with the Cytoscape analyzer tool identified the network with a confidence score of 0.699 and PPI enrichment *p* ≤ 1.0e−16, stating that the network has significant interactions beyond expectations. Also, central nodes were determined with criteria of interaction numbers of more than 10 nodes (Table 2). Finally, according to the interaction numbers of hubs and altered expression levels, ELANE, ARG1, DEFA4, and LCN2 were selected for further experimental analysis.

### 3.3 Analysis of LCN2, DEFA4, ELANE, and ARG‐1 expression levels

**Figure 5:**
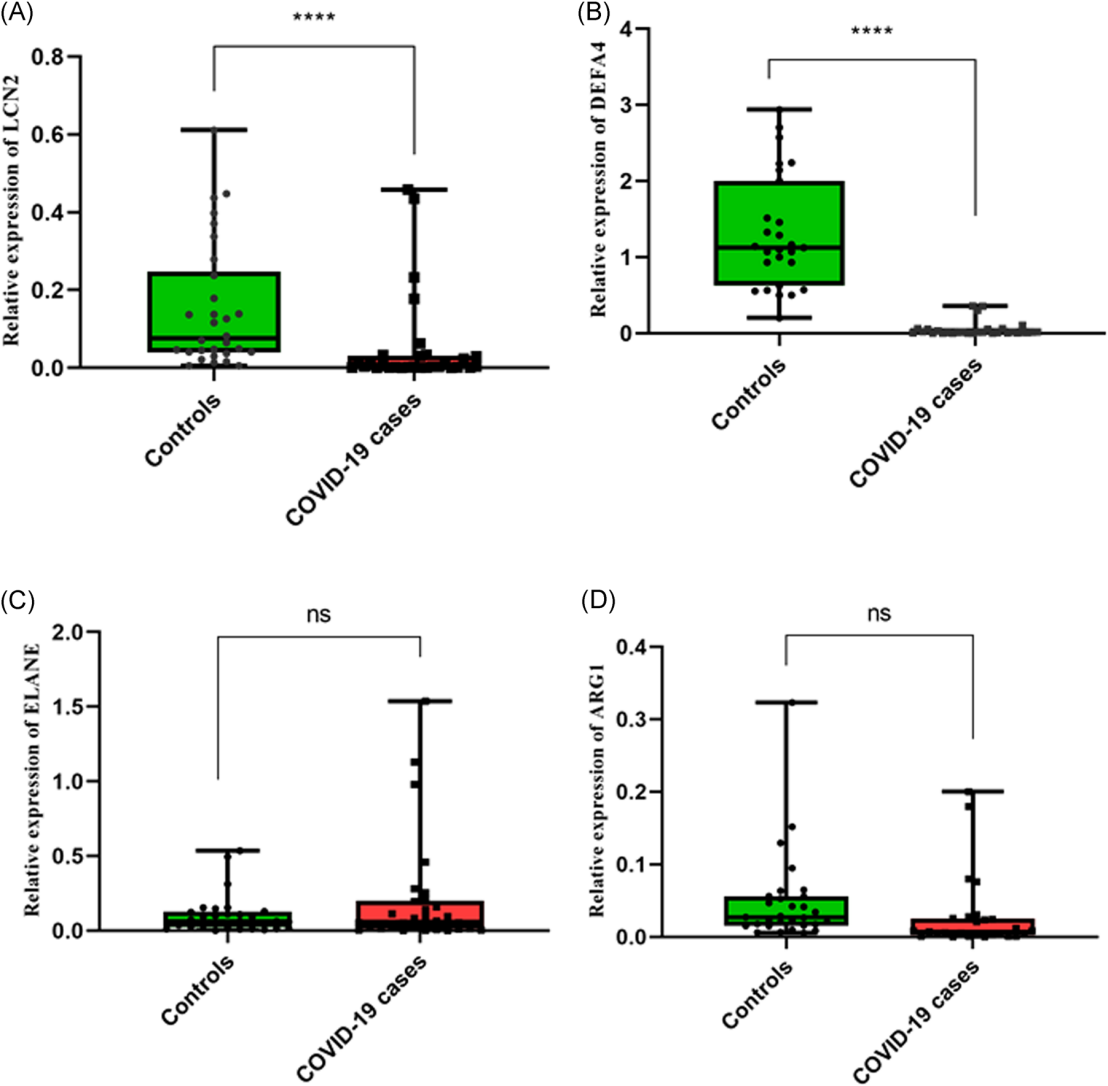
Quantitative real‐time polymerase chain reaction (PCR) analysis of LCN2 (A), DEFA4 (B), ELANE (C), and ARG‐1 (D) in COVID‐19 patients and control subjects. The expression levels of selected genes in whole blood samples of 30 COVID‐19 patients and 30 control subjects were evaluated. *p* Values are calculated by parametric *t* test (*p* value: 0 ≤ 0001, 0 ≤ 0001, 0.2, and 0.08, respectively). Glyceraldehyde‐3‐phosphate dehydrogenase was utilized as an internal reference to normalize mRNA levels

The results demonstrated that the relative expression of LCN2 and DEFA4 in COVID‐19 patients was significantly downregulated (logFC: −2.9, −5.35, respectively) compared with healthy individuals (Figure 5A,B). However, no significant difference in the ELANE and AGR‐1 expression levels (logFC: 0.97, −0.63) was found between COVID‐19 and control (Figure 5C,D).

## 4 DISCUSSION

Despite great efforts to develop effective vaccines, COVID‐19 treatment is still a critical issue worldwide. There has been a great effort upon detailed illumination of the underlying mechanisms and pathogenesis of the SARS and Middle East respiratory syndrome (MERS). In addition, searching for novel and efficient strategies of prevention and treatment of SARS and MERS has been the subject of intense interest since their onset in 2003 and 2012, respectively. Despite recent advances in the development of efficient vaccines, coronavirus still is a major threat to global health. Therefore, further studies are strongly required to provide detailed insights into the COVID‐19 pathogenesis. In this study, we focused on the in silico analysis of the irregular gene expression and biological processes that are considered to be vital in coronavirus pathogenesis. We selected SARS and influenza data sets to find DEGs in SARS‐positive and H1N1‐positive patients. Accordingly, common DEGs between the two mentioned data sets were identified and considered as hubs. SARS‐CoV‐2 is highly similar to the Influenza A virus in terms of transmission methods, clinical manifestations, and host immune responses to infection. Moreover, coinfection with SARS‐CoV2 and influenza virus has been reported in several patients.^20^ On the other hand, investigations have reported about 80% of the high protein homology sequence identities between SARS‐CoV and SARS‐CoV2,^21^ which may justify their selection. As confirmed by our results, neutrophil‐mediated immunity is the most significant biological process involving hubs. Our findings are consistent with a study by Didangelos et al. that reported significant downregulation of neutrophil genes in bronchoalveolar lavage fluid cells of COVID‐19 patients.^22^ The exact role of neutrophils during viral infections is not fully understood. Along with neutrophil antiviral defense function, degranulation, and lysis of neutrophils might be cytotoxic in pathological states such as severe coronavirus‐induced pneumonia as well as exacerbation of lung inflammation caused by the influenza virus. In addition, an increase in the ratio of neutrophils to peripheral lymphocytes is observed in some severe COVID‐19 cases, which might be associated with an unfavorable prognosis. Lung damage in some COVID‐19 patients might be due to neutrophil dysfunction.^22^ Following in silico studies, four genes, including ELANE, ARG‐1, LCN2, and DEFA4, were selected for further experiments. Real‐time PCR was applied to evaluate the altered expression of these DEGs in whole blood samples of COVID‐19 patients compared with healthy control subjects. The results determined that the expression levels of LCN2 and DEFA4 in COVID‐19 patients were significantly downregulated compared to the control group. However, no significant differences were observed in the expression of ELANE and ARG‐1. Our results determined a significant increase in the studied hubs in SARS‐positive and H1N1‐positive patients (logFC > 2, adj *p* ≤ 0.05). Therefore, these experimental findings are in conflict with bioinformatics results and other studies that demonstrate upregulated expression of neutrophil‐related genes in viral infections, including coronavirus. LCN2 plays a key role in immune responses.^23^, ^24^ Increased levels of LCN2 expression are associated with activation of the innate and humoral immune response, acute inflammatory response, cilium movement, and neutrophil migration.^25^, ^26^ LCN2 can inactivate macrophages and reduce inflammatory responses, leading to the devastating consequences of pneumococcal pneumonia. Overexpression of LCN2 in respiratory syncytial virus infection has been reported to be a very severe viral infection.^27^ DEFA4 is mainly expressed in neutrophils to increase virus uptake.^28^ However, these hubs could recruit inflammatory cells and participate in all phases of innate and adaptive immune responses in the lung that include initial deterioration of pathogens, mounting, and resolution. Furthermore, anti‐inflammatory effects of the investigated are reported as well. Recent evidence suggests that secreted α‐defensins could prohibit various respiratory viruses, including RSV, adenovirus, and parainfluenza virus.^28^ This antiviral effect could serve a novel field in the development of novel therapeutic strategies for viral infections.^29^, ^30^, ^31^ ARG1 expression in myeloid cells has emerged as a prominent regulator of innate and adaptive immune responses. In addition to wound healing properties, ARG1 activity can also suppress the antiviral immune response during some viral infections.^32^ ELANE is another important gene that is overexpressed in COVID‐19 patients. Neutrophils are known to be part of inflammatory responses that secrete elastin during viral infection. Increased elastase activity during viral infections could result in detrimental outcomes in pulmonary injury associated with the pathogenesis of chronic obstructive pulmonary disease, cystic fibrosis, ARDS, and pulmonary fibrosis.^33^, ^34^ Decreasing neutrophil load and increasing host defenses due to ELANE inhibition propose this system to protect the lungs in severe SARS‐CoV patients. Accordingly, timely control of cytokine storms in the early stages of virus entry is the basis for improving the treatment of COVID‐19 patients.^35^ A proteomic analysis study performed by Akgon et al., identified that ELANE was significantly increased in nasopharyngeal samples compared to the control group.^13^ One possible justification for controversial results could be explained by antibiotic effects on COVID‐19 patients. As previously mentioned, the patients who participated in this study were treated with antibiotics during their hospitalization period. Since the onset of COVID‐19, antibiotics have been prescribed to prevent bacterial coinfection in these patients. Besides, antibiotic consumption could reduce inflammatory response during pneumonia,^36^ suggesting that it could be an effective way to alleviate inflammation in COVID‐19 patients. Moreover, as discussed, neutrophils are considered to play a crucial role in SARS‐CoV‐2 infections. Evidence suggests the increased neutrophils in the respiratory tract during infection with influenza A virus and SARS‐CoV.^37^ The potential of ciprofloxacin and moxifloxacin to interact with COVID‐19 protease was assessed through an in silico study. Molecular docking results confirmed that ciprofloxacin and moxifloxacin bind more strongly to the active site of COVID‐19 protease than native ligands.^38^ Considering the predisposition of COVID‐19 to concomitant bacterial infection, Sharifipour et al., examined endotracheal aspirate samples of COVID‐19 patients admitted to the ICU. To identify bacterial strains, samples were cultured on bacterial media. Endotracheal aspirate samples were also collected and cultured on different media to support bacterial growth. Bacterial infections of *Acinetobacter baumannii* and *Staphylococcus aureus* were detected in all 19 patients. Based on antimicrobial susceptibility testing, *A. baumannii* strains were resistant to the evaluated antibiotics without Metallo‐beta‐lactamases producing except Colistin. One strain of *S. aureus* was resistant to methicillin while the other strain was sensitive to tested drugs that were identified as methicillin‐sensitive strain. Further investigations are required to confirm our findings in COVID‐19 patients coinfected with bacteria.^39^ In addition, a high resistant rate of *A. baumannii* strains to piperacillin, imipenem, ceftriaxone, ciprofloxacin, and ceftazidime are reported previously.^40^ It is determined that the activity of elastase in human neutrophils, as well as *Pseudomonas aeruginosa*, could be stimulated by Colistin and erythromycin while ceftazidime, tobramycin, and gentamicin might inhibit neutrophil elastase activity. Increased elastase activity by colistin is involved with the development of airways cystic fibrosis.^41^ A similar bioinformatics study by Hemmat et. al analyzed the GSE1739 microarray data set. Data were achieved by examination of the PBMCs of 10 SARS‐positive patients and four healthy subjects. Based on GEO2R analysis, the most important genes involved in SARS‐CoV infection are ELANE, ORM2, RETN, BPI, ARG1, DEFA4, CXCL1, and CAMP. In addition, the important biological process in the SARS infection refers to the activation and degranulation of neutrophils based on GO analysis. Neutrophilia, basophilia, and lymphopenia were also detected in infected patients. Prescription of Serpins and Arginase inhibitors have been suggested to increase survival during SARS‐CoV infection.^12^ Ultimately, our findings confirm that neutrophil activation and degranulation are mainly observed in SARS and H1N1 infection processes and that receiving the antibiotics could affect neutrophil activity during viral infection. However, it is being suggested that antibiotics could decrease inflammation by restoring the neutrophil‐related gene activity in COVID‐19 patients.

## 5 CONCLUSION

In conclusion, this investigation suggests that deregulated DEGs in viral infections such as SARS and influenza belong to innate immune components, particularly neutrophils. In addition, prescribing antibiotics to SARS‐CoV‐2 patients may decrease inflammation and pneumonia by restoring neutrophil‐related genes. However, to confirm these results and to clarify the treatment and prevention of SARS‐CoV‐2 infection, further studies in larger population sizes. In addition, it could be suggested for subsequent experiments to analyze gene expression in pulmonary neutrophils in severe COVID‐19 patients and compare with levels in peripheral blood neutrophils.

## CONFLICTS OF INTEREST

The authors declare no conflicts of interest.

## ETHICS STATEMENT

The informed written consent was obtained from the patient's parent or legal guardian. This study was approved by the Ethics Committee of Isfahan University of Medical Sciences, Isfahan, Iran, with ethics code IR.MUI.MED.REC.1399.439. Written informed consent was obtained from each patient before treatment.

## AUTHOR CONTRIBUTIONS

*Methodology, software, formal analysis, and writing—original draft*: Seyyed R. Mousavi. *Writing–review and editing*: Hajie Lotfi. *Resources, methodology*: Sharareh Salmanizadeh, Sara Feizbakhshan, and Farinaz Khosravian. *Resources, methodology, and software*: Maryam S. Sajjadi. *Resources and sampling*: Sajad R. Komachali. *Sampling*: Faeze A. Beni. *Methodology*: Banafshe Torkan and Mohammad Kazemi. *Resources and methodology*: Ramin Sami. *Conceptualization, validation, visualization, and supervision*: Mansoor Salehi. All authors have read and approved the final version of the manuscript. Salehi had full access to all of the data in this study and takes complete responsibility for the integrity of the data and the accuracy of the data analysis.

## Supporting information

Supporting information.

## Data Availability

The authors confirm that the data supporting the findings of this study are available within the article.
