# Supplementary figures and images for: An experimental in silico study on COVID‐19: Response of neutrophil‐related genes to antibiotics
Source: Health Sci Rep. 2022 Mar 7;5(2):e548. doi: 10.1002/hsr2.548 (PMC8900978; doi:10.1002/hsr2.548)

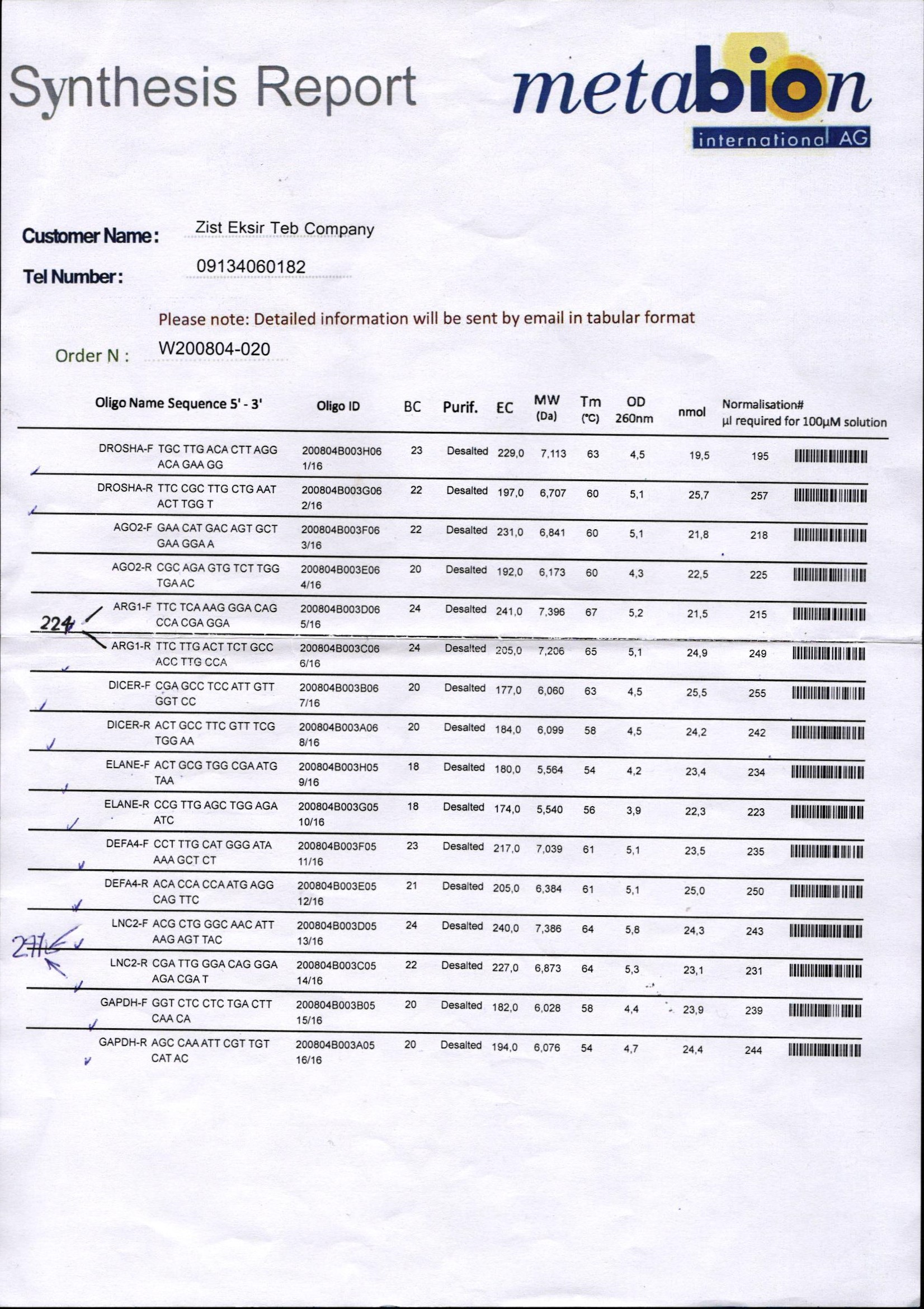

Supplement: Supplementary file 1 — Supporting information. [file HSR2-5-e548-s001.jpg]
